# Stiffness estimation of planar spiral spring based on Gaussian process regression

**DOI:** 10.1038/s41598-022-15421-1

**Published:** 2022-07-02

**Authors:** Jingjing Liu, Noor Azuan Abu Osman, Mouaz Al Kouzbary, Hamza Al Kouzbary, Nasrul Anuar Abd Razak, Hanie Nadia Shasmin, Nooranida Arifin

**Affiliations:** 1 Centre for Applied Biomechanics, Department of Biomedical Engineering, Faculty of Engineering, University of Malaya, 50603 Kuala Lumpur, Malaysia; 2 The Chancellery, Universiti Tenaga Nasional, 43000 Kajang, Malaysia

**Keywords:** Engineering, Mechanical engineering, Mathematics and computing

## Abstract

Planar spiral spring is important for the dimensional miniaturisation of motor-based elastic actuators. However, when the stiffness calculation of the spring arm is based on simple beam bending theory, the results possess substantial errors compared with the stiffness obtained from finite-element analysis (FEA). It deems that the errors arise from the spiral length term in the calculation formula. Two Gaussian process regression models are trained to amend this term in the stiffness calculation of spring arm and complete spring. For the former, 216 spring arms’ data sets, including different spiral radiuses, pitches, wrap angles and the stiffness from FEA, are employed for training. The latter engages 180 double-arm springs’ data sets, including widths instead of wrap angles. The simulation of five spring arms and five planar spiral springs with arbitrary dimensional parameters verifies that the absolute values of errors between the predicted stiffness and the stiffness from FEA are reduced to be less than 0.5% and 2.8%, respectively. A planar spiral spring for a powered ankle–foot prosthesis is designed and manufactured to verify further, of which the predicted value possesses a 3.25% error compared with the measured stiffness. Therefore, the amendment based on the prediction of trained models is available.

## Introduction

The outstanding performance of elastic actuators facilitates the wide application in various fields. Especially in the fields of exoskeletons, walking robots and powered upper- or lower-limb prostheses, a rotating elastic actuator with a sufficiently small size is desired. The key to dimensional miniaturisation lies in the design and performance of the elastic element, that is the torsion spring. When the required stiffness and twisting angle of the spring are large, two common kinds of torsion springs, the traditionally cold-coiled torsion spring and the torsion bar, are not suitable for miniaturisation. The former needs a large diameter of the material, correspondingly, large inner and outer diameters. The latter needs a long length. The research of planar torsion spring possesses the potential to solve the problem.

A typical design of the planar torsion spring is based on Archimedean spiral, usually called planar spiral spring, and the kind of springs has been engaged in different applications. A unidirectional series elastic actuator employs a planar spiral spring with a single spring arm^1^. A similar spring is designed and applied in a cycling-enhanced knee exoskeleton^2^. In the rehabilitation instruments, two motor-based series elastic actuators of gait rehabilitation^3,4^ and a hydro-elastic actuator for upper limb rehabilitation^5^ are designed with the planar spiral spring with two spring arms. Other kinds of planar torsion springs are also reported in several fields, such as humanoid robots^6^ and powered transfemoral prostheses^7^.

For a motor-based elastic actuator, the stiffness of the elastic element, which has an influence on the bandwidth of the control system and other performances, is an important objective during the design process. However, in the above applications of planar spiral springs, most of them fail to obtain the theoretical stiffness from the verification. For example, Lagoda et al.^3^ find that, for their designed spring, the actual spring stiffness differs considerably (219,000 Nmm/rad) from what has been calculated and simulated (353,000 Nmm/rad) based on simple beam bending equations. With a similar calculation method, Chaichaowarat et al.^2^ report that the geometry design obtained from the calculation shows more than two times stiffness than that of the design target. In the design from Stienen et al.^5^, the proposed geometry design and theoretical stiffness cannot be verified correctly in the finite-element analysis (FEA).

Several previous studies try a few methods to calculate the stiffness of planar spiral spring more accurately. A mathematical model based on curved beam theory is derived to describe the relationship between the stiffness and any given angular displacement of the spring, and a topological optimisation on the spring arm is achieved^8,9^. However, several of the parameters at the end of the spring arm in the derived equation, including the components of deformation forces, the components of displacement, and the differential coefficient of the arm deformation angle, are not easy to attain during the design process, which makes the calculation complicated and not suitable for the initial design. The other study discusses the influence of the number of springs, the turns of Archimedean spiral, the angle shift of inner support, and the spinning angle of assembly on the stiffness of spring^10^. However, the results are obtained from the FEA of limited models instead of a method of calculation or prediction. Lin et al.^11^ introduce an experienced correction coefficient determined by the external connection mode and circle number of the spring in the stiffness expression of the spring during the parametric modelling of the planar spiral spring. However, the problem of how to select the value of the experienced correction coefficient is not solved. Therefore, studying how to calculate the stiffness of the spring accurately and simply, which contributes to reducing the number of design iterations, is needed for the design and application of planar spiral spring.

To fill up the above research gap, utilising the prediction capacity of the Gaussian process regression (GPR), which is an excellent machine-learning solution for the problem with small samples and nonlinearity, may be able to amend the current method. GPR has shown its advantages in various fields. Gheytanzadeh et al.^12^ successfully estimate 13 kinds of metal–organic framework (MOF)-based adsorbents’ abilities of CO_2_ adsorption by GPR with a small amount of experimental data, in which the four kinds of input parameters have an apparent nonlinear relationship with the target. Based on a GPR model improved by the integrated radial basis function based kernel principal component analysis, Kong et al.^13^ construct a real-time, accurate tool wear predictive model between 48 kinds of signal features and the flank wear width of tool inserts. The study verifies that the improved GPR can deal with high-dimensional input vectors with complex relationships because all the signal features are extracted from time-domain, frequency-domain, and wavelet-domain, respectively. West et al.^14^ utilise the capacity of interpolation and extrapolation of GPR with sparse, highly clustered data to produce smoother estimates of radiation intensity in the reconstruction of nuclear radiation map when provided with uncertain, irregularly spaced observations. The fields of the applications of GPR include but are not limited to environment^15–17^, materials^18^, engineering^19,20^, and finance^21^. For the research target of this paper, the available data also express the above features, such as small samples, complex interrelationships and irregular observations. Therefore, GPR is selected as a possible solution, and the application of GPR in the field of mechanical design is explored.

After identifying which term is the source causing error mainly in the stiffness calculation, this paper contributes to the amendment of the error-source term via the prediction of GPR models, which are trained by finite-element data sets and corresponding geometrical parameters of Archimedean spiral spring arms or complete planar spiral springs. The amended method improves the accuracy of theoretical stiffness calculation when the calculated results are compared with the stiffness from FEA and the actual stiffness obtained by measuring the sample of the designed spring. The application of the amended method substantially improves the efficiency of designing a planar spiral spring and reduces the number of design iterations.

## Methods

### Stiffness estimation of spring arm

**Figure 1:**
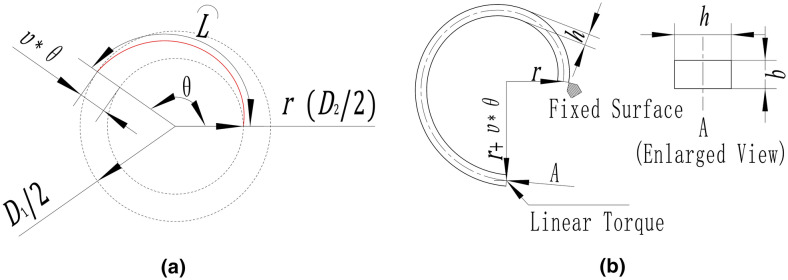
Parameters of spring arm and setting for FEA.

The spring arm is the main part of a planar spiral spring, and its geometry is based on Archimedean spiral, whose parametric equations are shown in Eq. (1).

1 $$\left\{\begin{array}{c}y=\left(r+\upsilon \times \theta \right)cos\theta \\ x=\left(r+\upsilon \times \theta \right)sin\theta ,\end{array}\right.$$

where $r$ is the spiral radius, $\upsilon$ is the spiral pitch and $\theta$ is the spiral wrap angle. A segment of Archimedean spiral (in red) with $\theta$ less than π is illustrated in Fig. 1a to explain the geometrical parameters. Spiral pitch ($\upsilon$) means the radial distance to the starting point increases $\upsilon$ mm when the spiral wrap angle increases 1 rad from the starting point. Therefore, the radial distance from the ending point to the starting point is $\upsilon *\theta$ mm. Based on these three basic parameters, the arc length of Archimedean spiral ($L$) can be estimated approximately by Eq. (2) or Eq. (3) with the inner and outer diameters of the spiral, ${D}_{1}$ and ${D}_{2}$, which are calculated in Eqs. (4) and (5).

2 $$L=\left[{\pi }^{2}\left({D}_{1}^{2}-{D}_{2}^{2}\right)\right]/(2\upsilon ),$$

3 $$L=[\theta ({D}_{1}+{D}_{2})]/4,$$

4 $${D}_{1}=2\left(r+\upsilon \times \theta \right),$$

5 $${D}_{2}=2r.$$

In previous literature^1–3,11^, the stiffness of the spiral spring arm is calculated via Eq. (6), which is similar to the stiffness calculation of hairspring based on simple beam bending theory^22^.

6 $${K}_{\mathrm{cal}\_\mathrm{a}}=EI/L=(Eb{h}^{3})/(12L),$$

where $E$ is the elastic modulus of material, $I$ is the moment of inertia of area, for a rectangular cross-section, $I=b{h}^{3}/12$, $b$ is the thickness and $h$ is the width, as shown in the enlarged view in Fig. 1b.

**Figure 2:**
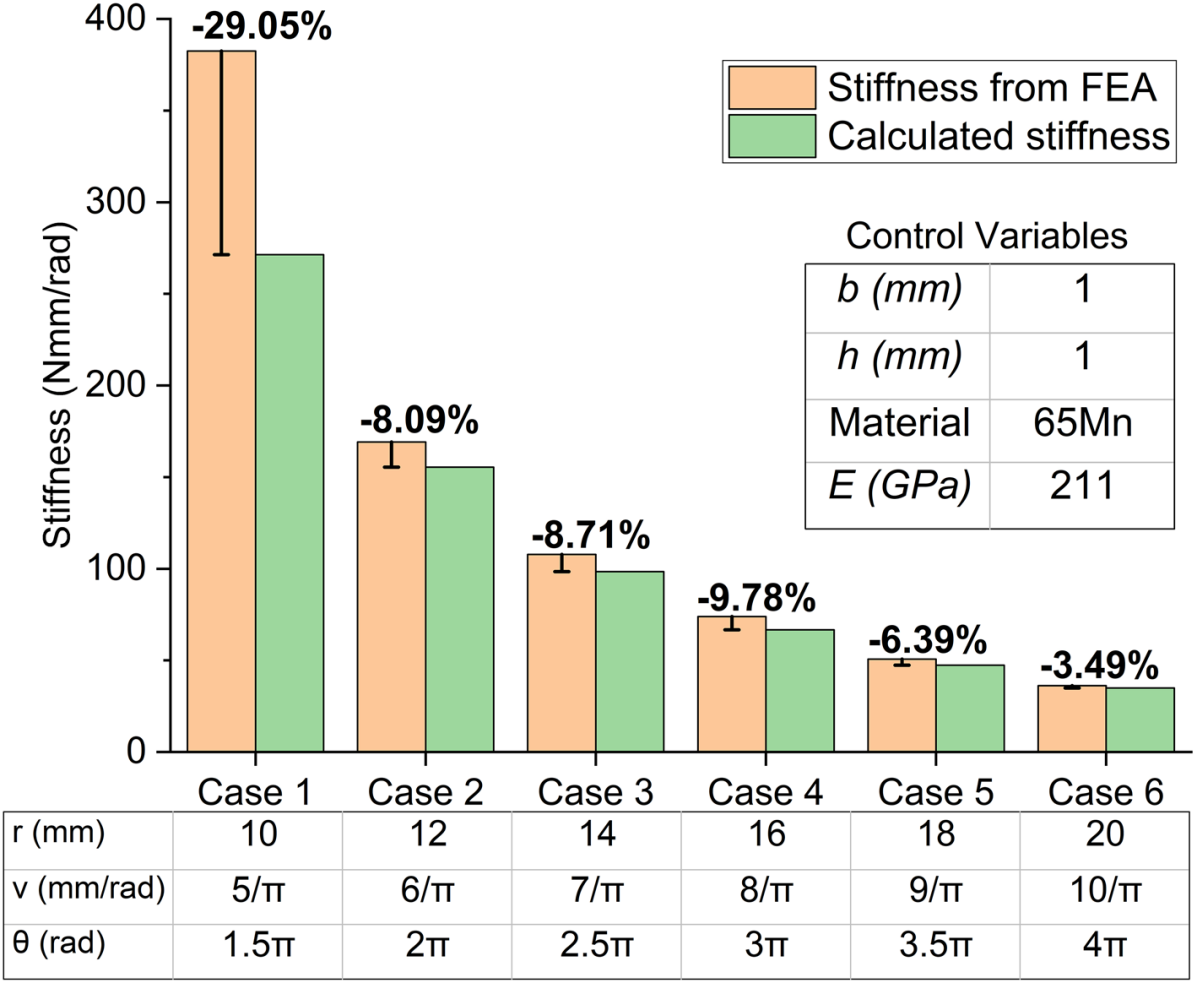
Comparison and parameters of six spring arm cases.

The $\theta$ value of the planar spiral spring is usually much smaller than the counterpart of the hairspring employed in the manufacture of a watch because of different application conditions. Therefore, as mentioned in the Introduction, the calculated results from the above equations cannot reflect the actual stiffness of the planar spiral spring to a large extent. To amend the current equation, firstly, it needs to identify whether the errors between the theoretically calculated results and the truth values show any general features amongst different cases. For this purpose, the stiffness of the spiral spring arms obtained from FEA, ${K}_{FE\_a}$, is introduced as the truth values to compare with the calculated stiffness (${K}_{cal\_a}$) from Eq. (6). In this paper, the stiffness from FEA refers in particular to the stiffness with minute deformation around the equilibrium position, and FEA is completed in ANSYS Workbench. Figure 1b shows that, in FEA, the cross-section of the end near the centre is set as a fixed surface, and the distal end is applied with a miniature linear torque, whose absolute value is from 0 to 0.1 Nmm, to rotate around the centre of the spiral. Torques in the positive and negative directions are applied, and the corresponding angular displacements of the distal end are obtained. Finally, when the intercept of fitting line is equal to 0, the fitting line slope of the linear regression between the torque versus the angular displacement is regarded as the value of the stiffness from FEA. Six cases of different segments of Archimedean spiral with arbitrary spiral parameters are employed as the basic centre line, and corresponding six spring arms are constructed with the same material and cross-sectional parameters (as the control variables), namely, elastic modulus of material ($E$), thickness ($b$), and width ($h$). The results of the calculated stiffness and the stiffness from FEA of the spring arms are shown in Fig. 2 with the parameters.

**Figure 3:**
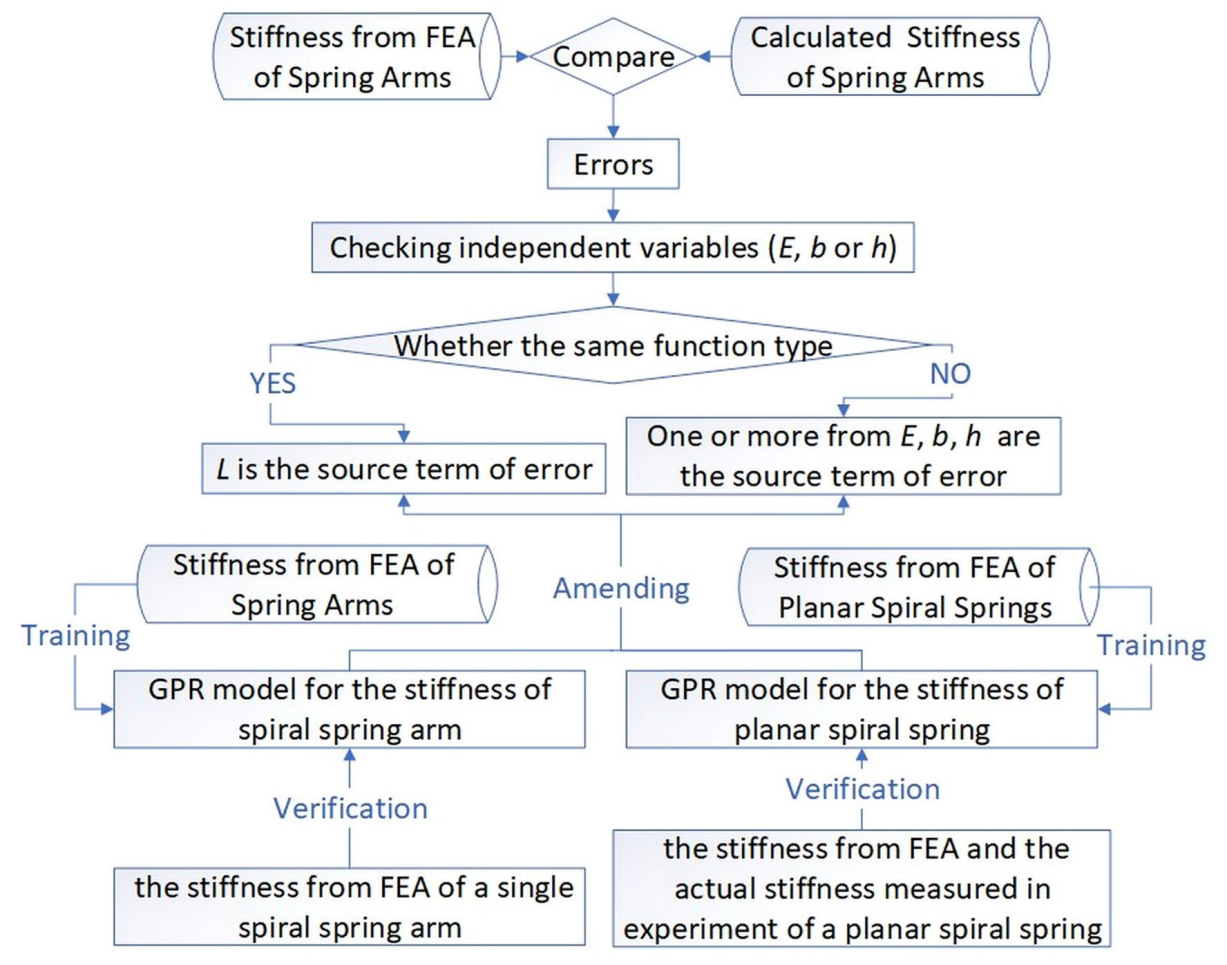
Flow chart of identifying the source term of error, training and verifying the GPR models.

In all cases, the calculated stiffness results are to varying degrees less than the stiffness from FEA, and the peak value of the error reaches − 29.05% in Case 1. However, no other apparent relationship between the value of error and the geometrical parameters of spring arms is found. Therefore, following the steps illustrated in Fig. 3, this paper identifies the source term of the error in the original Eq. (6), and then engages the method of GPR to amend the source term of error.

For the identification of the source term of error, the method is briefly explained as follows. Elastic modulus of material ($E$), thickness ($b$), and width ($h$) are set as the independent variable one by one, and the two other variables are the control variables. The unary function relation of the independent variable versus the dependent variable (the corresponding stiffness from FEA of the spring arms with the same spiral parameters from Case 1 to 6) is checked and compared with the known relation of the independent variable versus the theoretical stiffness from the calculation in the original Eq. (6). If the function types of the two relations, such as directly proportional function or power function, are different, the tested independent variable causes the error between the stiffness from FEA and the calculated stiffness. If all the three material or cross-sectional parameters keep the same function relation types with the stiffness from FEA as that described in the original Eq. (6), that is ${K}_{cal\_a}\propto E$, ${K}_{cal\_a}\propto b$ and ${K}_{cal\_a}\propto {h}^{3}$, the errors stem from the term of the arc length of Archimedean spiral ($L$) because the function from Eq. (6) includes only four parameters.

**Figure 4:**
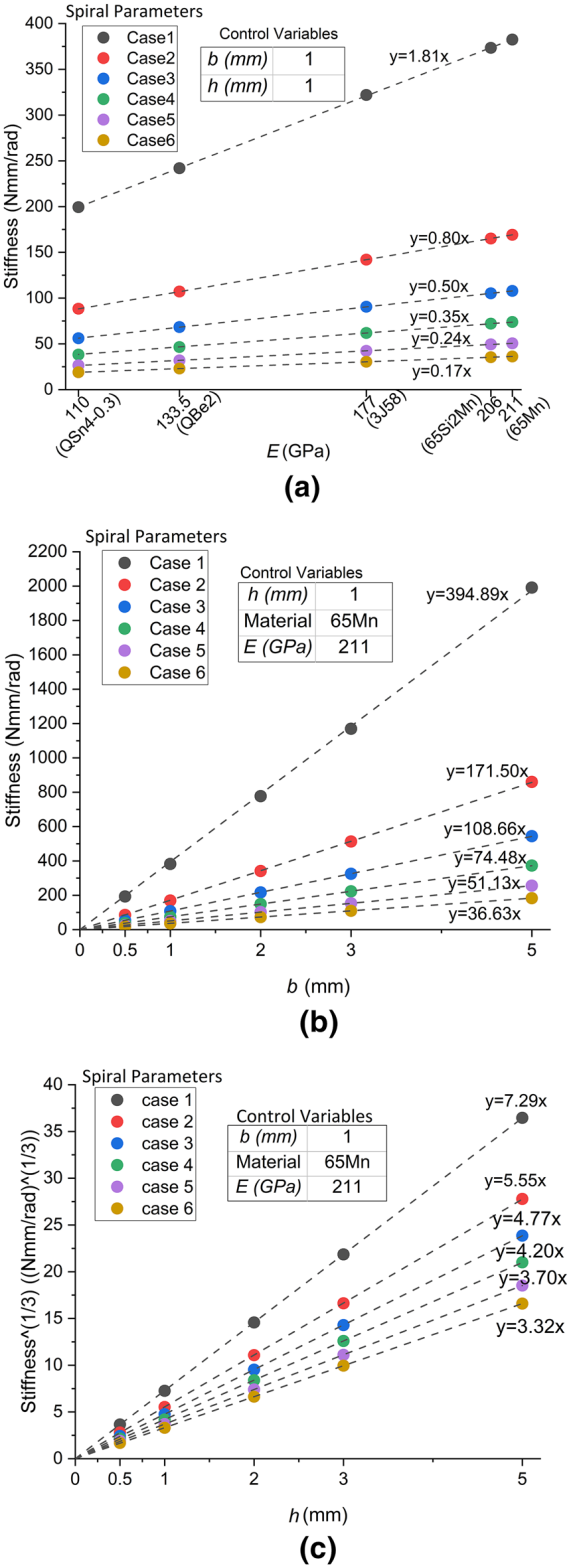
Relation between the stiffness from FEA of spring arms and the elastic modulus, thickness and width. (**a**) Stiffness from FEA versus elastic modulus of materials; (**b**) Stiffness from FEA versus thickness; (**c**) Stiffness from FEA versus width.

According to classical elasticity theory, the above errors are less likely to come from the parts of the elastic modulus and the cross-sectional parameters. However, these parameters of the spring arms are still analysed one by one, lest the unknown shear deformation, where the anisotropic modules and the equivalent moment of inertia of area should be considered, influences the calculation. With the same spiral parameters from Case 1 to 6 shown in Fig. 2, the corresponding stiffness from FEA of spiral spring arms versus different independent variables are illustrated in Fig. 4. The dash lines and the formulas in Fig. 4 show that the results of linear regression of each group of points. The intercepts of these fitting lines are controlled as 0, which ensures that all the dash lines go through the origin of coordinates, that is, the dependent variable has a directly proportional relation with the corresponding independent variable. Figure 4a shows that the data points of the stiffness from FEA almost coincide with the fitting lines with errors in the range of − 0.49 to 0.62%, which demonstrates that the stiffness from FEA is almost perfectly directly proportional to the elastic modulus of materials, that is ${K}_{FE\_a}\propto E$. Similarly, the directly proportional relation of ${K}_{FE\_a}\propto b$ is also demonstrated in Fig. 4b with an average error of 0.52%. In Fig. 4c, the value of the vertical coordinates is the cube root of the stiffness from FEA ($\sqrt[3]{{K}_{FE\_a}}$). With the average error of − 0.28%, the directly proportional relation between $\sqrt[3]{{K}_{FE\_a}}$ and width ($h$) is demonstrated, which is equivalent to ${K}_{FE}\propto {h}^{3}$. Therefore, according to Eq. (6), that $L$ is the source term of error is deduced.

Undoubtedly, for the arc length of Archimedean spiral, the difference between the estimated value from Eqs. (2) or (3) and the truth value obtained from the line integrals with respect to arc length is not substantial enough to generate the above errors between the stiffness from FEA and the calculated stiffness of spiral spring arms. In addition, because all the above errors are negative, one possible reason is that not all sufficiently small subsegments of Archimedean spiral work with identical elastic features during deformation, that is, not all the complete arc length of Archimedean spiral is effective in the operation of the spiral spring arm. Therefore, this paper hypothesises that an effective working length (${L}_{e}$), which could be used to amend the original term of the arc length of Archimedean spiral (the source term of error) in Eq. (6), may exist in each different configuration of spiral spring arms. It is worth noting that the definition of the effective working length in this paper is only a possible assumption instead of a strict definition in elastic mechanics, and the value is obtained from the inverse calculation via the data of the stiffness from FEA of the spiral spring arms with the material and the cross-sectional parameters.

Based on the front view shown in Fig. 1b, when a value of $h$ is defined, the geometrical shape in this view of the spring arm is only decided by the three spiral parameters of Archimedean spiral, namely, $r$, $\upsilon$ and $\theta$. In this case, the proposed effective working length (${L}_{e}$) is also decided with the defined geometrical shape of the spring arm. As a consequence, ${L}_{e}$ is a function of $r$, $\upsilon$ and $\theta$, as follows.

7 $${L}_{e}(r, \upsilon , \theta )=(Eb{h}^{3})/(12{K}_{FE\_a}).$$

With controlling variables of the material (65 Mn, $E=\mathrm{211,000 MPa}$), thickness ($b=1\mathrm{ mm}$) and width ($h=1\mathrm{ mm}$), 216 spring arms are modelled with the full combination of the three independent variables, whose domains of definition are $r \in \left\{10, 12, 14, 16, 18, 20\right\}$ in mm, $\upsilon \in \left\{5/\pi , 6/\pi , 7/\pi , 8/\pi , 9/\pi , 10/\pi \right\}$ in mm/rad, and $\theta \in \left\{1.5\pi , 2\pi , 2.5\pi , 3\pi , 3.5\pi , 4\pi \right\}$ in rad.

After the values of stiffness are analysed by FEA, the effective working lengths (${L}_{e}$) are calculated. With the obtained 216 data sets of {${L}_{e}, r, \upsilon , \theta$}, a GPR model is trained with the method of fivefold cross-validation in the application of Regression Learner from MATLAB (MathWorks). GPR models are nonparametric kernel-based probabilistic models that explain the response through a Gaussian process and explicit basis functions and predict new responses according to Bayesian inference^23,24^. The detailed mathematical principle of GPR is analysed in reference^23^, and the brief deduction can refer to literature^12^ and website^24^. When the optimisable GPR is selected to train in the Regression Learner (MATLAB), the software will optimise five kinds of parameters, including basis function, kernel function, kernel scale, sigma and standardising. Thereinto, for the basis function, the software searches among zero, constant and linear. For the kernel function, the options include five major categories: rational quadratic, squared exponential, Matern 5/2, Matern 3/2 and exponential models, and each category also includes nonisotropic and isotropic models. All the mathematical expressions of the above kernel functions can be found in the introduction of Kernel (Covariance) Function Options from the website of MathWorks^25^.

The trained GPR model can mimic the unknown function and generate the estimate of the effective working length ($\widehat{{L}_{e}}$) of a spiral spring arm, whose parameters belong to any subset of $\left\{r\in {\mathbb{R}}|10\ll r\ll 20\right\}$, $\left\{\upsilon \in {\mathbb{R}}|5/\pi \ll \nu \ll 10/\pi \right\}$ and $\left\{\theta \in {\mathbb{R}}|1.5\pi \ll \theta \ll 4\pi \right\}$. Based on the predicted estimate of effective working lengths ($\widehat{{L}_{e}}$), the Eq. (6) can be amended by substituting $\widehat{{L}_{e}}$ to replace the original term of the arc length of Archimedean spiral ($L$). In this case, Eq. (8) can be used to estimate the stiffness of the single spring arm.

8 $$\widehat{{K}_{\mathrm{a}}}=EI/\widehat{{L}_{e}}.$$

### Stiffness estimation of planar spiral spring

When a planar spiral spring is designed, it usually includes more than one identical spring arm to increase total stiffness. If only considering the number of spring arms is $n$, it is easy to verify that the total stiffness is $n$ times the stiffness of a single spring arm according to theory of parallel springs.

9 $${K}_{\mathrm{cal}\_\mathrm{s}}=n{K}_{\mathrm{cal}\_\mathrm{a}}=nEI/L.$$

**Figure 5:**
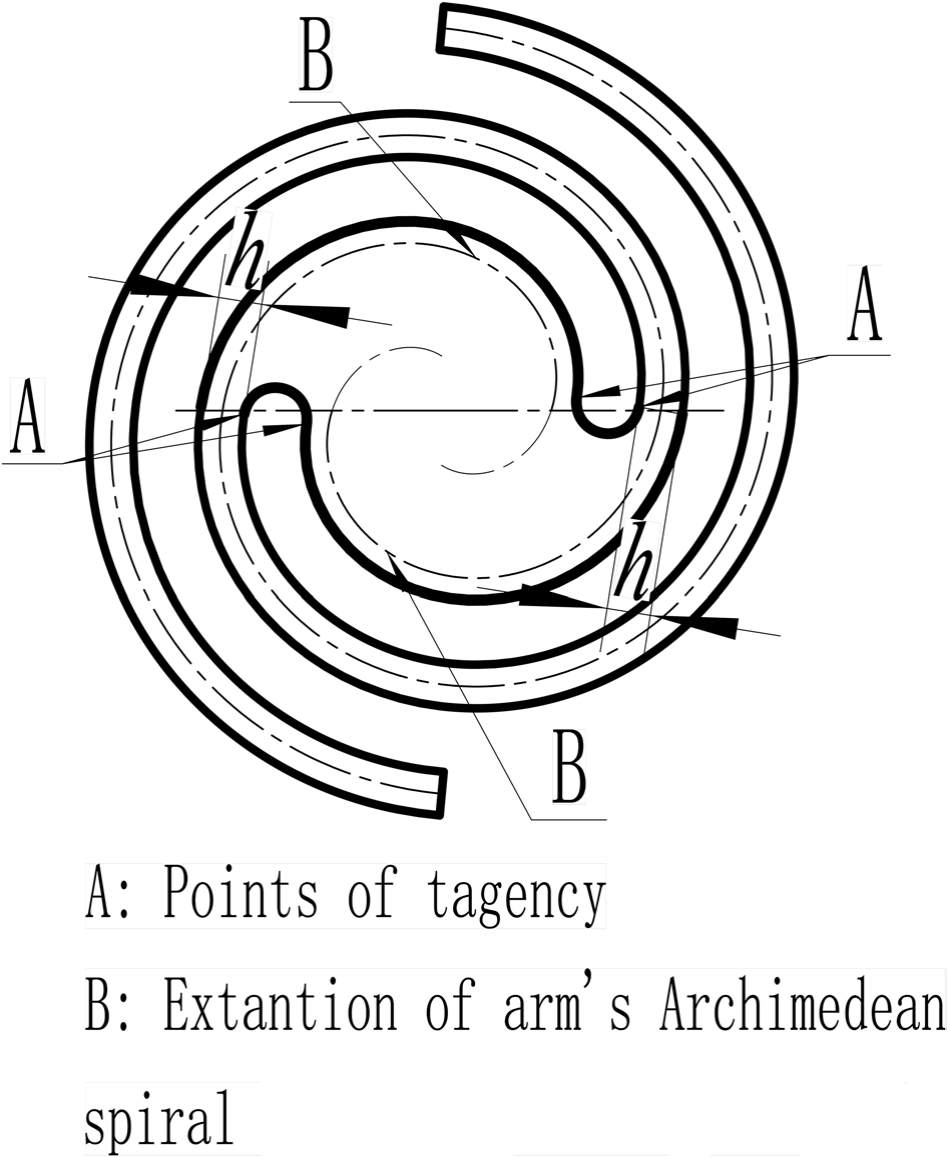
Example of planar spiral spring with two spring arms and its geometrical relationships.

However, including a connecting part amongst the spring arms is necessary to assemble the spring in the actual design. An example of the connecting part is designed to combine two identical spring arms, and its geometrical relationships of features are shown in Fig. 5. The selected Archimedean spiral centre lines, which start from $\theta =0$ in the positive direction, are offset in both directions with the distance equal to half of the width ($h$/2) to form the profiles of the spring arms. The dash-pot lines with tag B are the reverse extension (from $\theta =0$ to $\theta =-1.5\uppi$) of Archimedean spiral, and offset to form the profile of the central connecting part. These two parts’ profiles are transited smoothly with half-circles at the points of A.

**Figure 6:**
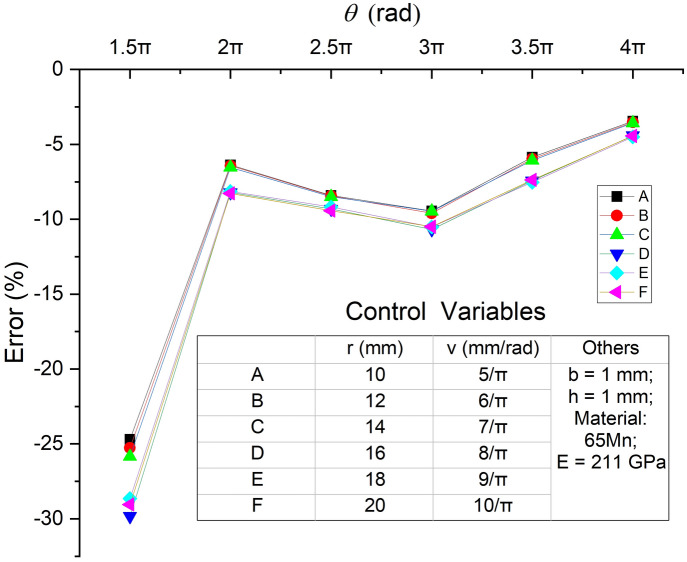
Errors between twice calculated stiffness and stiffness from FEA of double-arm planar spiral springs.

The combination of Figs. 2 and 5 shows that the geometrical shape of a planar spiral spring in the view of Fig. 5 is defined by four parameters, namely, $r$, $\upsilon$, $\theta$ and $h$. This paper preliminarily analyses the influence of the wrap angle ($\theta$) of Archimedean spiral on the accuracy of the calculated stiffness of planar spiral springs. Six groups of double-arm planar spiral springs with arbitrary parameters are constructed with the same method in Fig. 5. Their data of the stiffness from FEA are compared with the twice values of the calculated stiffness of the corresponding spring arms from Eq. (6). The errors and the parameters are illustrated in Fig. 6. The group of $\theta =1.5\pi$ has much more substantial errors than the other groups, which are in the range of − 24 to − 29%. In addition, this work will design a planar spiral spring to verify the accuracy of the amendment for the theoretical stiffness calculation. The spring requires meeting the requirement of applying in a powered ankle–foot prosthesis, which will be introduced later. According to the preliminary estimation of the space for the assembly of the designed spring, it is less likely able to allow the planar spiral spring with a large wrap angle. To sum up the above two reasons, this work only studies the influence of the connecting part on the planar spiral spring when the spring arms have a spiral wrap angle $\theta =1.5\pi$.

Based on the above hypothesis about effective working length (${L}_{e}$), the connecting part’s influence on complete spring stiffness is converted into the variation of the effective working length of each spiral arm ($\Delta L$). The definition of $\Delta L$ is also only a possible assumption instead of a strict definition in elastic mechanics. With a constant wrap angle $\theta =1.5\pi$, for any prescribed spiral parameters ($r$ and $\upsilon$) and the width of the spring arm ($h$), the geometrical shape of the connecting part is defined. Therefore, $\Delta L$ is a function of $r$, $\upsilon$ and $h$. $\Delta L$ can be obtained from Eq. (10).

10 $$\Delta L\left(r,\upsilon ,h\right)=(nEb{h}^{3})/(12{K}_{FE\_s})-{L}_{e},$$

where $n$ is the number of spring arms, and $n$=2 in this paper.

With the same material (65 Mn, $E=\mathrm{211,000 MPa}$), thickness ($b=1\mathrm{ mm}$), spiral wrap angle ($\theta =1.5\pi$), total 180 planar spiral springs are modelled with the method in Fig. 5, with the full combination of the three independent variables, whose domains of definition are $r \in \left\{10, 12, 14, 16, 18, 20\right\}$ in mm, $\upsilon \in \left\{5/\pi , 6/\pi , 7/\pi , 8/\pi , 9/\pi , 10/\pi \right\}$ in mm/rad, and $h\in \left\{1, 1.5, 2, 2.5, 3\right\}$ in mm.

In FEA, for each modelled planar spiral spring, a minute hole is made at the centre of the connecting part to work as the fixed surface with the negligible influence on the stiffness, and the same linear torque in the analysis of the spring arm is set to drive both distal ends of the two spring arms together. With the stiffness from FEA (${K}_{FE\_s}$) data, the values of $\Delta L$ are calculated. The second model of GPR is trained with the data sets of {$\Delta L$, $r$, $\nu$, $h$} to predict the estimate of the variation of the effective working length ($\widehat{\Delta L}$) of a planar spiral spring, whose parameters belong to any subset from $\left\{r\in {\mathbb{R}}|10\ll r\ll 20\right\}$, $\left\{\upsilon \in {\mathbb{R}}|5/\pi \ll \nu \ll 10/\pi \right\}$, and $\left\{h\in {\mathbb{R}}|1\ll h\ll 3\right\}$. The Eq. (11) is obtained to estimate the stiffness of the two-arm planar spiral spring by substituting $(\widehat{{L}_{e}}+\widehat{\Delta L}$) into Eq. (9) to replace $L$.

11 $$\widehat{{K}_{\mathrm{s}}}=nEI/(\widehat{{L}_{e}}+\widehat{\Delta L}).$$

This paper takes two steps to verify the above two trained GPR models. First, several spring arms and springs with given arbitrary dimensional parameters are modelled to get the stiffness from FEA and compared with the estimated results from Eq. (8) and Eq. (11). Second, to achieve a specific stiffness, a planar spiral spring for a series elastic actuator of a powered ankle–foot prosthesis is designed by Eq. (11). Similarly, the stiffness from FEA of the designed spring arm and the planar spiral spring are analysed. The designed planar spiral spring is also manufactured, and its physical stiffness is measured in the test of torque versus rotating angle. Results are converted into the equivalent stiffness of the unit thicknesses ($b=1\mathrm{ mm}$), which is convenient to compare the results with the stiffness estimates to verify the accuracy of the predictions.

According to our previous study, the external torque load on a sound ankle can be obtained from analysing the kinematics of the ankle joint and the ground reaction force during walking on level ground. According to the maximal capacity of the designed powered ankle–foot prosthesis, a torque load is obtained from a subject who weighs 100 kg. Applying the torque load to the inverse dynamic model of the series elastic actuator of the prosthesis, the speed requirement of the actuator can be optimised by a proper stiffness of the series elastic element, that is, the planar spiral spring, with selected parameters of motor and transmission. This paper does not discuss many details of calculation. The result of minimising the speed requirement of the actuator shows that the stiffness of the planar spiral spring needs to reach the value of 350,000 Nmm/rad, and the maximum of positive rotating angle needs to be at least 25°. On the aspect of the spring’s geometrical design, within the dimensional limit of $\mathrm{\varnothing }60\mathrm{ mm}\times 45\mathrm{ mm}$, the smaller dimensional size is, the better the reduction in the volume and the weight of the whole device.

## Results

**Figure 7:**
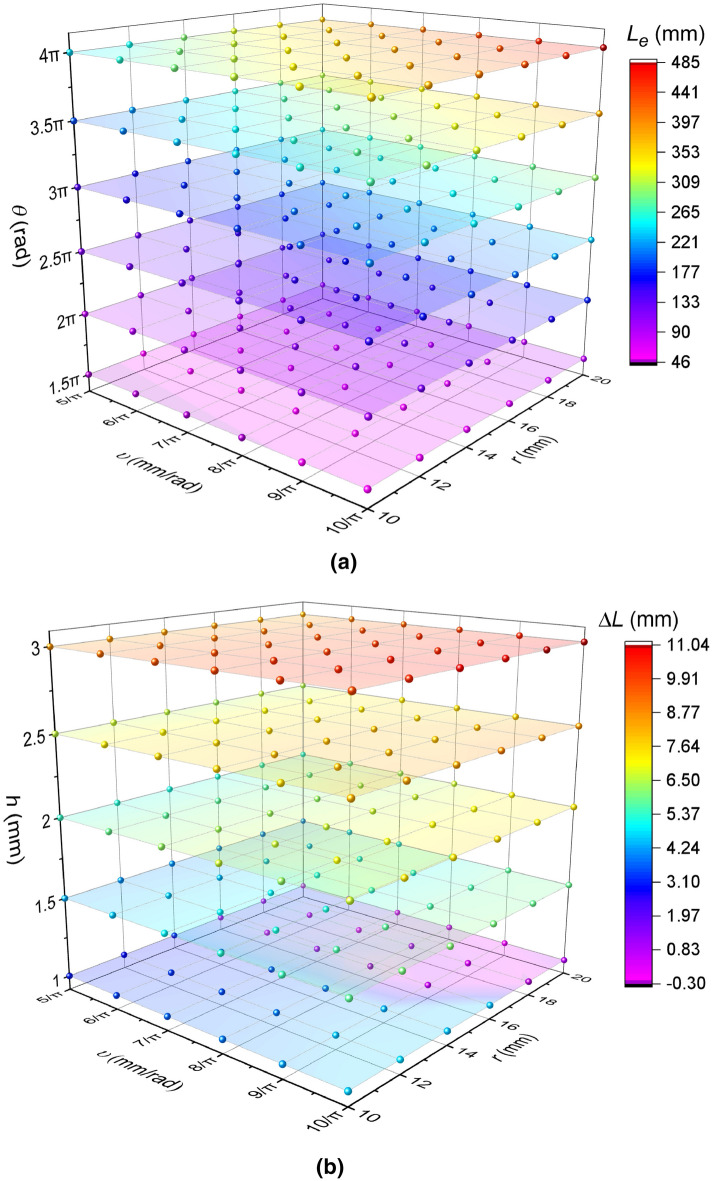
Results of effective working lengths and variation of effective working length of each spiral arm. (**a**) Effective working lengths of each spiral arm (${L}_{e}$); (**b**) Variation of effective working length of each spiral arm ($\Delta L$).

The relationship between the calculated effective working lengths (${L}_{e}$) of the spring arms versus the parameters of spiral radius ($r$), spiral pitch ($\upsilon$) and spiral wrap angle ($\theta$) is shown in Fig. 7a. Within each plane of $\theta$, ${L}_{e}$ increases as the increase of $r$ and as that of $\upsilon$, and the increasing rates are almost the same. Amongst the planes of $\theta$, a larger value of $\theta$ results in a higher increasing rate of ${L}_{e}$ as the increase of $r$ or $\upsilon$. Compared with the calculated spiral length ($L$) from Eq. (2) or (3), the percentage of the difference value occupied $L$, that is (${L}_{e}-L$)/$L$%, is in the range from − 37.54 to − 1.84%. However, the relationship between the difference and the other parameters is not clear.

Figure 7b illustrates the relationship between the spring samples’ variation of the effective working length of each spiral arm ($\Delta L$) versus the parameters of spiral radius ($r$), spiral pitch ($\upsilon$) and width ($h$). Amongst planes of $h$, a larger $h$ is more likely to contribute to a larger variation ($\Delta L$) and a larger rate of variation ($\Delta L/{L}_{e}$) because the ${L}_{e}$ of each plane of $h$ is the same as one another and equal to that of the plane of $\theta =1.5\uppi$ in Fig. 7a. Moreover, the relationship between $\Delta L$ and {$r$, $\upsilon$} almost presents the characteristic that, when $\upsilon$ is less and $r$ is larger, the $\Delta L$ is less than the counterpart of the larger $\upsilon$ and the less $r$. In other words, from the current view, the value of $\Delta L$ becomes larger from the distal vertex to the proximal vertex in each plane of $h$. However, the characteristic is not strict.

Two models of GPR are trained with the above results. The optimised hyperparameters of trained models are listed in Table 1.

**Table 1:** Optimised hyperparameters of trained Gaussian process regression models.

	1st Model of {${L}_{e}, r, \nu , \theta$}	2nd Model of {$\Delta L, r, \nu , h$}
Basis function	Constant	Constant
Kernel function	Nonisotropic matern 5/2	Nonisotropic exponential
Kernel scale	1.4247	0.41212
σ	0.009315	0.00063918
Standardise	true	false

**Figure 8:**
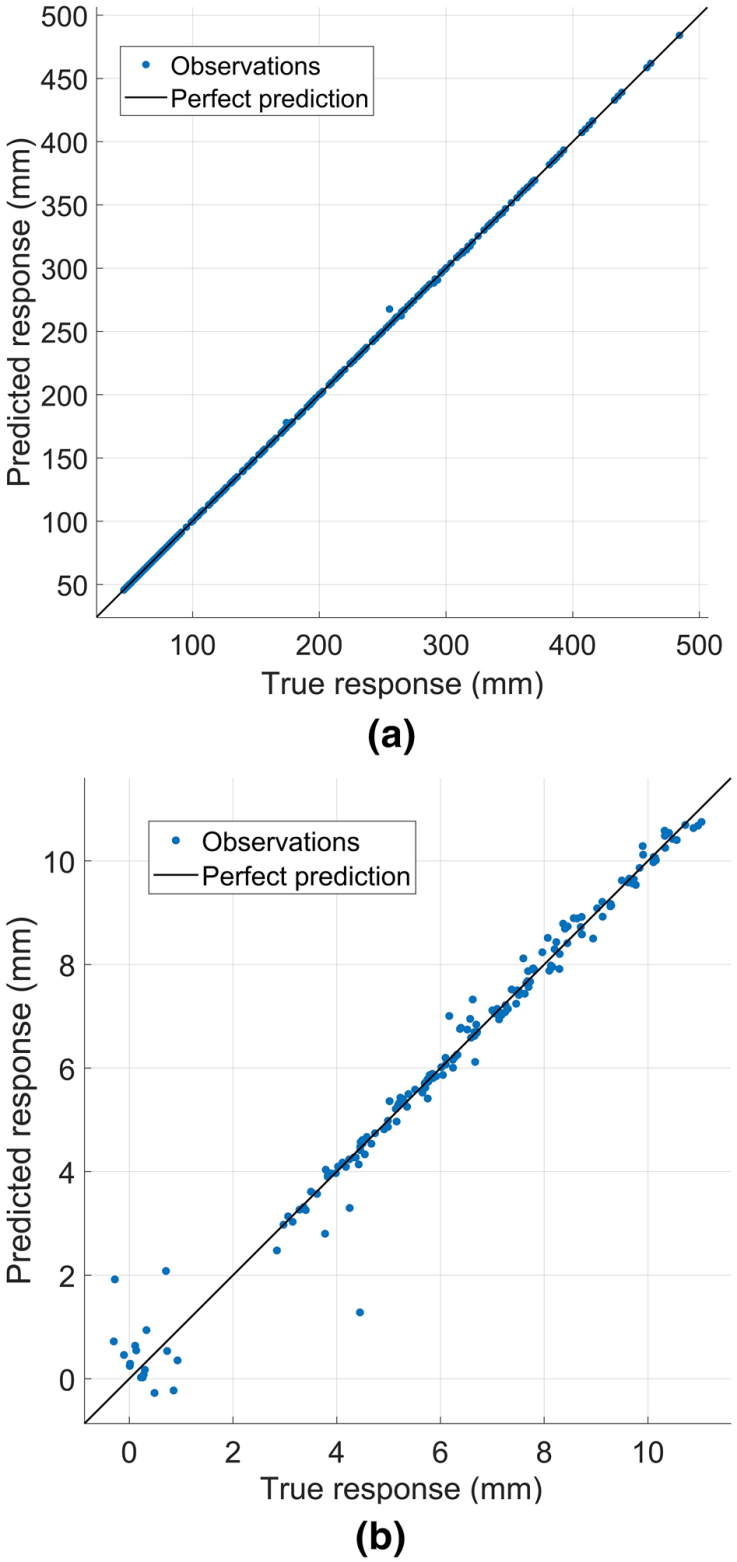
Predicted and true responses of effective working lengths and variation of effective working length of each spiral arm. (**a**) Effective working lengths of each spiral arm (${L}_{e}$); (**b**) Variation of effective working length of each spiral arm ($\Delta L$).

The predicted and actual plots of the above models are shown in Fig. 8, and the value of R-squared (difference between the true response and the predicted values) is used to evaluate the precision of the model. The R-squared value is in the range of 0–1. If the value is closer to 1, the precision is better, and vice versa. In Fig. 8a, almost all the predicted responses of the effective working lengths (${L}_{e}$) overlap with the true responses, and the R-squared with the value of 1 is achieved. In Fig. 8b, when the values of $\Delta L$ are small, and especially when the values are negative, the predicted responses of the variation of the effective working length of each spiral arm ($\Delta L$) possess substantial residuals. However, the overall tendency still tracks the line of perfect prediction, and the value of R-squared is 0.99.

On the first step of verifying the trained GPR models, five spring arms and five two-arm planar spiral springs are engaged for the 1st and the 2nd GPR models, and their material and dimensional parameters and corresponding comparison results of stiffness are listed in Tables 2 and 3, respectively. All the dimensional parameters are generated randomly. In Table 2, the errors between the calculated stiffness from Eq. (6) and the stiffness from FEA are in the range of − 7.84 to − 9.99%. In comparison, the errors between the predicted stiffness by the 1st GPR model and the stiffness from FEA are reduced to the range from − 0.01 to 0.48%, and it is worth noting that there are four samples whose absolute values of errors are less than 0.10%. In Table 3, although the performance of the 2nd GPR model is not as good as the 1st GPR model, the amendment from the trained GPR model to the original calculation is effective. According to Eq. (9), the absolute values of errors compared with the stiffness from FEA are more than 18%. The trained 2nd GPR model reduces the errors to be less than 2.8%. Therefore, it is verified that the trained GPR models are able to predict the stiffness of single spring arms and the two-arm planar spiral springs more accurately, respectively.

**Table 2:** Parameters and stiffness comparison of spring arms.

	Case 1	Case 2	Case 3	Case 4	Case 5
Material: 65 Mn; $E=\mathrm{211,000 MPa}$; $b=1\mathrm{ mm}$;$h=1\mathrm{ mm}$					
$r$ (mm)	18.46	15.59	13.48	12.26	10.51
$\upsilon$ (mm/rad)	8.74/π	9.82/π	5.44/π	6.36/π	5.88/π
$\theta$ (rad)	3.13π	1.99π	1.85π	2.47π	3.06π
Calculated stiffness from Eq. (6): ${K}_{cal\_a}$ (Nmm/rad)	55.64	110.90	163.43	112.65	93.77
Estimate stiffness: $\widehat{{K}_{a}}$ (Nmm/rad)	61.37	123.20	178.19	123.65	104.10
Stiffness from FEA: ${K}_{FE\_a}$ (Nmm/rad)	61.38	123.18	177.34	123.72	104.17
Error: ${(K}_{cal\_a}-{K}_{FE\_a})/{K}_{FE\_a}$	− 9.35%	− 9.97%	− 7.84%	− 8.95%	− 9.99%
Error: $(\widehat{{K}_{a}}-{K}_{FE\_a})/{K}_{FE\_a}$	− 0.01%	0.01%	0.48%	0.06%	− 0.07%

**Table 3:** Parameters and stiffness comparison of two-arm planar spiral springs.

	Case 1	Case 2	Case 3	Case 4	Case 5
Material: 65Mn; $E=211000\mathrm{ MPa}$; $b=1\mathrm{ mm}$; $\theta =1.5\mathrm{\pi rad}$;$n=2$					
$r$(mm)	18.15	11.77	14.34	16.85	15.71
$\upsilon$(mm/rad)	6.34/π	9.44/π	8.41/π	7.87/π	5.36/π
$h$(rad)	2.12	2.78	2.43	1.26	1.71
Calculated stiffness from Eq. (9): ${K}_{cal\_s}$(Nmm/rad)	3104.33	8505.76	5186.11	656.10	1891.26
Estimate stiffness: $\widehat{{K}_{s}}$ (Nmm/rad)	3937.01	11,213.00	6741.28	887.43	2403.81
Stiffness from FEA: ${K}_{FE\_s}$ (Nmm/rad)	3832.22	10,923.17	6615.73	864.47	2386.47
Error:${(K}_{cal\_s}-{K}_{FE\_s})/{K}_{FE\_s}$	− 18.99%	− 22.13%	− 21.61%	− 24.10%	− 20.75%
Error:$(\widehat{{K}_{s}}-{K}_{FE\_s})/{K}_{FE\_s}$	2.73%	2.65%	1.90%	2.66%	0.73%

**Figure 9:**
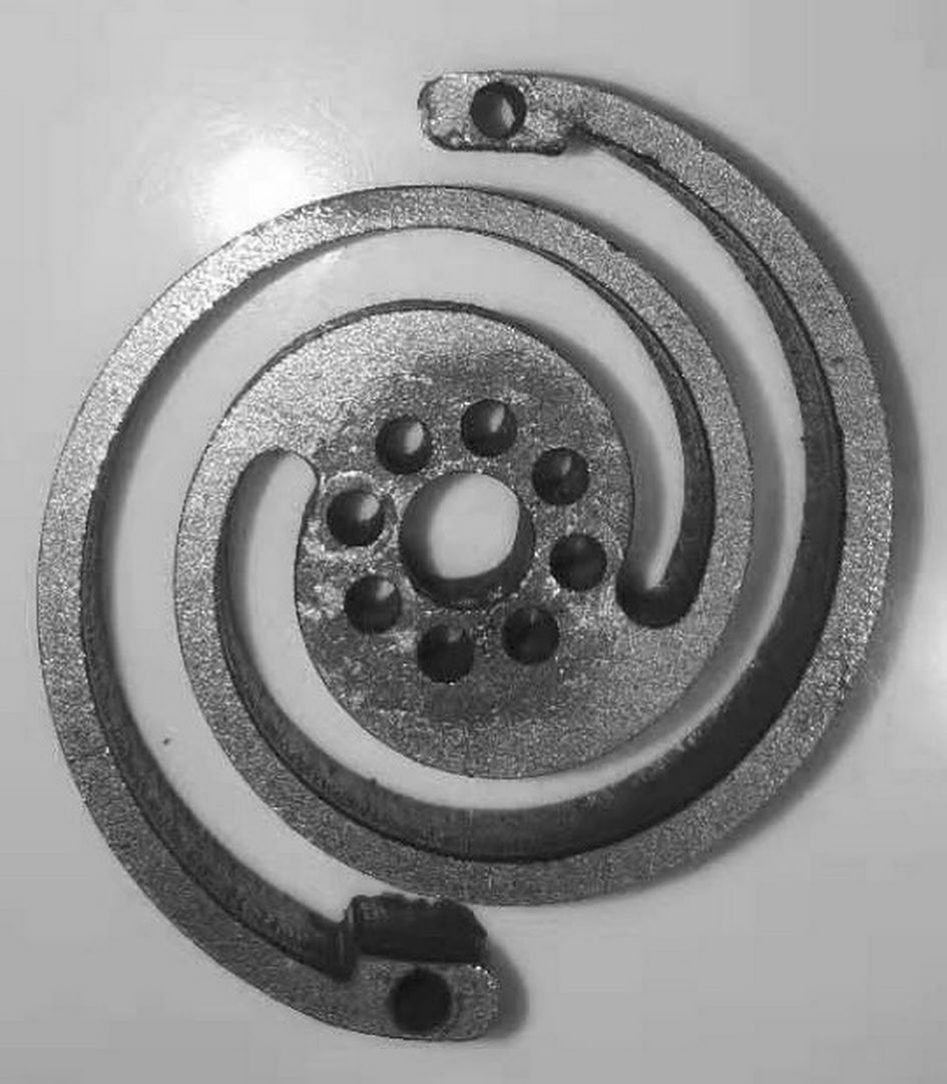
Manufactured planar spiral spring.

In further verification, given specific stiffness and dimensional requirements, a design of a planar spiral spring is completed according to the estimated results output by both trained models via several iterations. The designed spring is manufactured and shown in Fig. 9. Table 4 lists the selected parameters of dimension, the estimated results and the verification results in unit thickness, that is $b=1\;\mathrm{ mm}$. For the complete spring and its corresponding single spring arm, the errors between the predicted stiffness and the stiffness from FEA are 1.13% and 0.20%, which are not out of the above ranges, respectively. In comparison with the physical stiffness of the complete spring measured in the experiments, the stiffness estimate from trained GPR models possesses a 3.25% error.

**Table 4:** Parameters of designed spring arm and corresponding planar spiral spring when thickness is in unit thickness ($b=1 \; \mathrm{mm}$).

Items	Value	Items	Value
Spiral radius $r$ (mm)	15.5	Spiral pitch $\upsilon$ (mm/rad)	7.5/π
Spiral angle $\theta$ (rad)	1.5π	Width $h$ (mm)	2.71
Material	18Ni(350) Maraging Steel	Estimate of variation of effective working length of each spiral arm $\widehat{\Delta L}$ (mm)	8.64
Elastic modulus (MPa)	200,000	Estimate stiffness of spring $\widehat{{K}_{s}}$ (Nmm/rad)	8393.62
Number of spring arms $n$	2	FE stiffness of spring ${K}_{FE\_s}$ (Nmm/rad)	8302.45
Estimate of effective working lengths $\widehat{{L}_{e}}$ (mm)	70.37	Error of spring between FE and predicted stiffness ($\widehat{{K}_{s}}-{K}_{FE\_s}$)/${K}_{FE\_s}$	1.13%
Estimate stiffness of single spring arm $\widehat{{K}_{a}}$ (Nmm/rad)	4714.09	Mean stiffness of manufactured spring ${K}_{s}$ (Nmm/rad)	8129.81
FE stiffness of single spring arm ${K}_{FE\_a}$ (Nmm/rad)	4704.53	Error of spring between physical and predicted stiffness ($\widehat{{K}_{s}}-{K}_{s}$)/${K}_{s}$	3.25%
Error of single spring arm ($\widehat{{K}_{a}}-{K}_{FE\_a}$)/${K}_{FE\_a}$	0.20%		

On the aspect of dimensional requirements, the outer diameter of the designed spring is approximately 56.5 mm considering the width of the connecting parts on both distal ends of spring arms, which is less than the requirement of 60 mm. Owing to the directly proportional relation between the stiffness and the thickness of spring, according to the estimated stiffness of spring $\widehat{{K}_{s}}$ in unit thickness, which is 8393.62 Nmm/rad, $b$ only needs 41.70 mm to obtain the total stiffness of 350,000 Nmm/rad. If according to the physical stiffness, $b$ needs up to 43.05 mm, which can also meet the design requirement.

**Figure 10:**
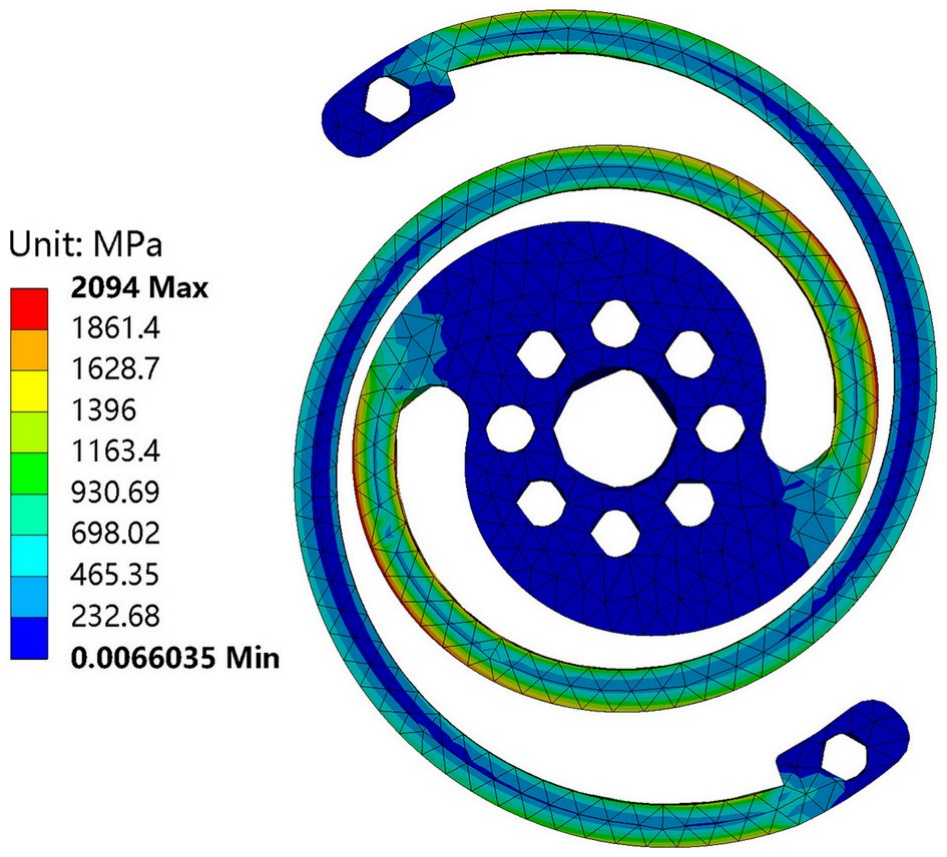
Von-Mises stress and deformation at rotated angle of 25° of designed spring.

On the last aspect of the operation scale, a linear rotating angle from 0° to 25° is applied to the designed spring model in FEA. Figure 10 shows the von-Mises stress and deformation results at the rotated angle of 25°. The maximum stress is below the yield tensile strength of the material of 18Ni(350) maraging steel, which is 2363 MPa. No intersection is between the spring arms stemming from the large deformation, demonstrating that the selection of dimensional parameters is reasonable.

## Discussion

This paper successfully applies the trained GPR models to amend the current stiffness calculation method of planar spiral spring. FEA is one of the most important methods to simulate and analyse the mechanical properties in the mechanical design process. Generally, although FEA results cannot be totally same as the experimental results because it is extremely difficult to simulate all actual conditions, the accuracy of FEA results is high enough. Therefore, this paper regards the stiffness from FEA as the truth value during error analysing. However, a disadvantage of FEA is that it will cost a long time for a complicated structure, which reduces the efficiency of iteration design. The original stiffness calculation is based on simple beam bending theory. However, as described above, its accuracy is low and random when the spiral wrap angle of a spring arm is small. Within the same theoretical framework, the proposed method in this paper solves the accuracy problem by amending the parameter of spiral length in the original equation via training GPR models with FEA data. Based on trained GPR models, the predicted parameters can be obtained without the FEA process, and the corresponding stiffness estimate can be calculated numerically. Although there are still some errors between the prediction of trained GPR models and the FEA, the trade-off between the accuracy and the processing time benefits the efficiency of the iteration design of a planar spiral spring to a large extent.

When the reason for the errors caused by the original stiffness calculation is analysed, the term of arc length in the original equation is identified as the source term of the error. Because the calculated stiffness is randomly less than the stiffness from FEA of the spring arms, it assumes that an effective working length may exist, which is less than the spiral’s arc length when the number of spiral turns is small. This phenomenon is possibly caused by the uncertain position of deformation in this kind of beam structure. In simple terms, the deformation does not evenly distribute throughout the whole structure, especially under small deformation or small load. This paper adopts a method of estimation based on the training GPR models with finite data sets to amend the original term of the arc length of Archimedean spiral for different objects in the design process intuitively and straightforwardly. For the spiral spring arm, the amendment is completed by replacing the arc length of Archimedean spiral with the assumed effective working length. For the complete planar spiral spring, based on the former, the variation of the effective working length of each spiral arm is added to the correction term to include the influence of the connecting part amongst spring arms. However, the method cannot disclose the essence of the uneven deformation, which needs further study in elastic mechanics. In addition, if the existence of the effective working length or that of the variation of the effective working length is verified theoretically, the values of different structures should be measured physically instead of inversely calculated.

From the perspective of the predicted results, compared with the calculated stiffness of the spring arm from the original equation, the first trained model has the capacity to reduce the absolute values of errors to less than 0.5%. Therefore, utilising the estimate of the assumed effective working length to replace the actual spiral’s arc length is an available amendment to the original equation. The amendment can also be applied to other cross-sectional shapes of the spring arm because of the directly proportional relation between moment of inertia of area and stiffness.

The performance of the second trained model is not as good as the first one on predicting the influence of the connecting part on the stiffness of planar spiral spring, but the errors are still acceptable compared with the stiffness from FEA (the values are less than 3%). The larger errors may come from the error accumulation. For the research of the connecting part, this paper only pays attention to the spring with two identical spring arms, whose spiral wrap angle is 1.5π rad. Therefore, several limitations are more likely in the direct application in other structures. However, the method of estimation is feasible with the data of different structures. In addition, when a planar spiral spring is designed, there are always some mounting holes on the connecting part for the assembly. The influence of mounting holes on the stiffness is also not considered in this paper because of the high uncertainty of the holes’ position distribution and sizes. Generally, the existence of mounting holes on the connecting part will increase the total stiffness to some extent because of the reduction of the deformable region.

Finally, during the verification with given specific stiffness, the error between the measured stiffness of the manufactured spring and the predicted stiffness is more substantial than any other errors (3.25%). However, the error is reasonable because the stiffness from FEA is set as the truth value instead of the real stiffness of the manufactured spring. Therefore, the error mainly comes from the difference between the stiffness from FEA and the real stiffness of the manufactured spring, which is caused by several possibilities. The machining error is a possible reason because it needs at least two methods, such as lathing and laser engraving, to manufacture such a piece of planar spiral spring, which creates the accumulation of machining errors. The second reason is the stiffness of assistive fixtures during the experiments, which is not included in the prediction. Similarly, the two mounting parts on the distal ends of spring arms are not analysed in the prediction. The last possibility is that the measurement accuracy of instruments is not high enough in the torque and angle measurement when the rotated angle of spring only occupies a small part of the total measuring range.

In a word, the amendment based on the trained GRP models in the stiffness calculation that is based on simple beam bending theory is available to improve the accuracy of the theoretical stiffness of the planar spiral spring and its spring arm, and the method can be applied to other kinds of springs. However, the terms employed to complete the amendment still need further theoretical verification in elastic mechanics. Finally, richer data are preferred for the training to improve the precision of the prediction in future work.

## Conclusion

This paper contributes to amending the stiffness calculation of the planar spiral spring based on Archimedean spiral by training two GPR models. The results find the term of arc length of Archimedean spiral is the source of the significant errors between the calculated stiffness and the stiffness from FEA of the spiral spring arms. Amending the original error-source term in the equation, the improved calculation can considerably reduce the error between the calculated stiffness of and the stiffness from FEA of the spiral spring arms and the complete planar spiral springs. The predicted theoretical stiffness also has an acceptable error compared with the actual stiffness obtained by measuring the piece of manufactured planar spiral spring. The application of the amended equation is able to improve the efficiency of designing a planar spiral spring and reduce the number of design iterations.

## Supplementary Information

Supplementary Information.
